# Signification of distal urinary acidification defects in hypocitraturic patients

**DOI:** 10.1371/journal.pone.0177329

**Published:** 2017-05-19

**Authors:** Valentina Forni Ogna, Anne Blanchard, Rosa Vargas-Poussou, Adam Ogna, Stéphanie Baron, Jean-Philippe Bertocchio, Caroline Prot-Bertoye, Jérôme Nevoux, Julie Dubourg, Gérard Maruani, Margarida Mendes, Alejandro Garcia-Castaño, Cyrielle Treard, Nelly Lepottier, Pascal Houillier, Marie Courbebaisse

**Affiliations:** 1 Centre d’Investigations Cliniques, Hôpital Européen Georges Pompidou, Assistance Publique-Hôpitaux de Paris, Paris, France; 2 Centre de Référence des Maladies Rénales Héréditaires de l’Enfant et de l’Adulte (MARHEA), Paris, France; 3 Université Paris Descartes, Faculté de Médecine, Paris, France; 4 Service de Génétique, Hôpital Européen Georges Pompidou, Assistance Publique-Hôpitaux de Paris, Paris, France; 5 INSERM CIC 14.29, Hôpital Raymond Poincaré, Assistance Publique-Hôpitaux de Paris, Garches, France; 6 Département de Physiologie, Unité rénale, Hôpital Européen Georges Pompidou, Assistance Publique-Hôpitaux de Paris, Paris, France; 7 Service d’ORL, Hôpital Bicêtre, Assistance Publique-Hôpitaux de Paris, Paris, France; 8 Université Paris-Saclay, Paris, France; 9 INSERM 1182, Paris, France; 10 Institut Necker Enfants-Malades, INSERM U1151 –CNRS UMR 8253, Paris, France; 11 INSERM UMR_S1138, CNRS ERL8228, Paris, France; University Jean MONNET of SAINT-ETIENNE, UNITED STATES

## Abstract

**Background and objectives:**

Hypocitraturia has been associated with metabolic acidosis and mineral disorders. The aim of this study was to investigate the occurrence of urinary acidification defects underlying hypocitraturia.

**Materials and methods:**

This retrospective observational study included 67 patients (32 men), aged 40.7±15.1 years with hypocitraturia (<1.67 mmol/24-h) and nephrolithiasis, nephrocalcinosis, and/or bone demineralization, referred to our center from 2000 to 2015. We aimed to assess renal distal acidification capacity, prevalence and mechanisms of urinary acidification defects. Patients with low baseline plasma HCO_3_^-^ (<22 mmol/L) were studied by bicarbonate loading or furosemide/fludrocortisone tests. Patients with normal baseline plasma HCO_3_^-^ had an ammonium-chloride challenge test. A normal response was a decrease in urinary pH <5.3 and an increase in urinary NH_4_^+^ ≥33 μmol/min and defined idiopathic hypocitraturia.

**Results:**

Eleven patients (16.4%) had low HCO_3_^-^ and overt distal acidification defect. Three had a mutation in the gene encoding AE1, 4 had Gougerot-Sjögren syndrome and no cause was found in the remaining 4 cases. Fifty-six patients (83.6%) had normal HCO_3_^-^; of those, 33 (58.9%) had idiopathic hypocitraturia. Among the 23 (41%) remaining patients, 12 were unable to increase urinary NH_4_^+^ excretion (among them, 8 were able to decrease urinary pH and 4 were not) whereas 11 were able to increase urinary NH_4_^+^ excretion but unable to decrease urinary pH. These 11 patients had higher fasting urinary calcium, reflecting bone resorption, than the other 12 patients: median 0.41 [0.24–0.47] vs. 0.22 [0.08–0.37] mmol/mmol creatinine (*P = 0*.*04*).

**Conclusions:**

Patients with hypocitraturia and normal plasma HCO_3_^-^ frequently show a latent acidification defect that can be further dissected into one of several subtypes based on urinary pH and NH_4_^+^ response to the acid load. Those patients with impaired urine acidification capacity but preserved NH_4_^+^ excretion exhibit particularly high calciuria and should be identified to optimize nephrolithiasis prevention.

## Introduction

Hypocitraturia is a risk factor for nephrolithiasis, since citrate is a potent inhibitor of the crystallization of stone forming salts [1] and has been associated with low bone density [2]. Once filtered, citrate is reabsorbed in the proximal tubule, so that only 10–35% of filtered citrate is excreted [3]. The urinary excretion rate of citrate is extremely sensitive to the acid-base balance [3]. Hypocitraturia is always present in patients with overt metabolic acidosis but can also reveal subtle defects in urinary acidification [3, 4]. These defects may be insufficient to cause an overt acidosis when the subject is in the steady state but may induce an acid-base imbalance that modifies citrate metabolism [5]. Whether hypocitraturia is a marker of a subtle defect in urinary acidification has never been established [6]. Distal renal tubular acidosis (dRTA) may result from either hereditary or acquired diseases. Inactivating mutations in the *SLC4A1* gene encoding the chloride bicarbonate exchanger AE1 are the main causes of autosomal dominant dRTA [7]. Loss of function mutations in *ATP6V1B1* and *ATP6V0A4*, genes encoding the B1 and a4 subunits of the apical H^+^-ATPase, are responsible for autosomal recessive dRTA, often associated with sensorineural deafness [6]. Acquired dRTA frequently develops as a consequence of Gougerot-Sjögren syndrome [8] and other autoimmune diseases [9, 10]. Very few studies have evaluated the utility of screening for a urinary acidification defects in non-acidotic patients with concomitant hypocitraturia, recurrent nephrolithiasis [11, 12], and/or bone demineralization [13, 14]. In the present study, we retrospectively analyzed data from patients with low citrate excretion and nephrolithiasis, nephrocalcinosis, and/or bone demineralization. We compared the clinical and biological characteristics of patients with overt acidosis to those of patients with normal acid-base status and here report results of acute acid load, genetic and immunological tests in hypocitraturic patients without overt acidosis. We develop hypotheses about the pathophysiological mechanisms underlying the subtle acidification defects that do not lead to overt acidosis.

## Materials and methods

### Patients

This study was conducted in accordance with the Declaration of Helsinki and approved by the French national regulatory board (CNIL, 915528 and 1922081). According to the French national regulatory board, all patients were informed before any exploration that their data could be used anonymously for clinical research. Given that the study was observational and only implied current care, they only had to give their oral informed consent. However, all patients gave written informed consent for biobanking (DC2009950) and, if required, specific written consent for genetic testing. The written consents for biobanking and genetic testing were recorded in the patient’s chart stored in our department and a copy was given to the patient

Patients were referred to our unit for the assessment of mineral disorders: recurrent kidney stones, nephrocalcinosis, and/or bone demineralization. Urinary citrate excretion is routinely measured in these patients.

In our retrospective analysis we included patients with low citrate excretion (<1.67 mmol/24-h), defining hypocitraturia [15], having undergone a renal acidification capacity test. Subjects with urinary bacterial contamination (≥10^5^ colonies/mL) or incomplete 24-hour urinary collection [16] were not included in the study because of the risk of falsely low urinary citrate excretion. We deliberately included patients with hypokaliemia, a confounding factor of hypocitraturia [3], since it is also a frequent finding in dRTA. Patients with overt metabolic acidosis (baseline venous plasma HCO_3_^-^<22 mmol/L), underwent a bicarbonate loading test [17] until 2009 and a furosemide/fludrocortisone test [18] thereafter. Patients with normal (≥22 mmol/L) baseline plasma HCO_3_^-^ underwent a short acid-loading test.

### Functional tests

The tests and analytical methods are fully described in the supplementary material (S1 File).

#### Bicarbonate loading test

Urine and venous blood samples were collected for measurement of pH and pCO_2_ at baseline and at hourly intervals for 4 hours during IV infusion of 1.4% sodium bicarbonate [17]. Maximal transport of HCO_3_^-^ should be >22 mmol/L of glomerular filtration rate (GFR) and, in the absence of urinary concentration defects, the urine-to-blood PCO_2_ difference should increase >20 mmHg during infusion [19].

#### Furosemide/fludrocortisone test

After plasma and urine sampling, 40 mg furosemide and 1 mg fludrocortisone were orally administered. Normal response is a decrease in urinary pH<5.3 at least once within 6 hours [18].

#### Acute acid loading test

Oral administration of 2 mmol/kg body weight ammonium chloride (NH_4_Cl) was performed after baseline plasma and urine sampling. Normal response is a decrease in urine pH<5.3 at least once and an increase in urinary NH_4_^+^ excretion ≥33 μEq/min. at least once within 6 hours [4]. We used both criteria to classify patients within four patterns of urinary acidification response: subjects able to acidify urine to a pH<5.3 and to increase urinary NH_4_^+^-excretion rate ≥33 μEq/min. ("idiopathic hypocitraturia"); patients unable to decrease urine pH but able to increase NH_4_^+^ ("high U.pH, high U.NH_4_"); patients able to acidify urine but unable to increase NH_4_^+^ ("low U.pH, low U.NH_4_"); patients unable to reduce the urine pH and to increase NH_4_^+^ ("high U.pH, low U.NH_4_").

#### Analytical methods

The pH and pCO_2_ in plasma and urine were measured using an automated pH and gas analyzer (ABL 555330 then 705; Radiometer, Copenhagen, Denmark). Plasma and urine HCO_3_^-^ concentration were calculated using the Henderson-Hasselbalch equation, with an α of 0.03, as follows: pH = 6.1 + log (total CO_2_ αPCO_2_); HCO_3_^-^ = total CO_2_-αPCO_2_.

Urine concentrations of NH_4_^+^ and TA were measured by titration [20, 21]. Calculation of net acid excretion (NAE) employed the standard formula [22]: NAE = NH_4_^+^ + TA−HCO_3_^-^. Percentage of acid load excreted was defined as the ratio of the total NAE after NH_4_Cl administration, divided by total NH_4_Cl load.

### Etiological screening

Genetic testing for *SLC4A1* mutation and missense polymorphism (p.Glu161Lys) of the *ATP6V1B1* gene was systematically proposed to patients with overt acidosis without evidence of an autoimmune disease and in patients with normal acid-base status if urinary pH remained >5.3 after acid load. Genetic testing for other mutations in *ATP6V1B1* and *ATP6V0A4* genes was performed only in patients with overt acidosis after exclusion of mutations in *SLC4A1* [23]. Sequencing methods are detailed in supplementary material (S1 File). If past or current clinical signs suggested an autoimmune disease, a biological screening including serum electrophoresis and antinuclear antibodies was performed. Sjogren’s syndrome was defined according to the American-European-Consensus-Group (AECG) criteria [24].

### Statistics

Statistical analyses were performed using STATA 12.0 (StataCorp) and R 3.1.2 statistical software (R Core Team 2014, GNU General Public License). Median and interquartile ranges (IQR) are used to describe continuous variables; numbers and frequencies to describe categorical variables. We used the Wilcoxon rank sum test and Chi-squared test to compare the characteristics of groups when appropriate, and the nonparametric Kruskas-Wallis test to assess differences in urinary acidification defect subgroups. Statistical significance was defined as *P*<0.05.

## Results

### Study population

From 2000 to 2015, 67 patients meeting the inclusion criteria were investigated.

Patients were first grouped based on the presence (N = 11, 16.4%) or the absence (N = 56, 83.6%) of an overt metabolic acidosis at baseline. In all the patients with overt metabolic acidosis, dRTA was confirmed using a bicarbonate-loading test (N = 5) or a furosemide/fludrocortisone test (N = 6).

When compared with patients without overt metabolic acidosis (Table 1,column 2), patients with overt metabolic acidosis (Table 1,column 1) were more frequently of female gender and more frequently had a nephrocalcinosis.

**Table 1:** Demographic and medical characteristics of the patients.

	Overt metabolic acidosis	Normal acid-base status
N	11	56
Age (years)	42 [21–47]	40 [29–50]
BMI (kg/m^2^)	22.0 [20.0–23.5]	23.7 [21.2–25.5]
Gender (female)	9 (82%)	26 (46%) *
Autoimmune disease	4 (36%)	6 (11%)
Nephrolithiasis	11 (100%)	50 (89%)
Nephrocalcinosis	4 (36%)	1 (2%) **
Low bone density—osteoporosis	4 (36%)	9 (39%)
RAAS inhibitors	0	6 (11%)
Diuretics	0	3 (6%)
Alkali therapy	1 (9%)	1 (2%)

Values are expressed as medians [interquartile range] or numbers (percentages) as appropriated. BMI: body mass index; RAAS: renin-angiotensin-aldosterone system: Autoimmune disease was defined as any previous or new diagnosis of autoimmune disease. Nephrolithiasis was defined as a previous history of kidney stones (confirmed by a radiologic detection or by the expulsion of a stone). Evaluation for low bone density-osteoporosis by DXA was performed in only 23 out of 56 patients with normal acid-base status. Low bone density-osteoporosis is defined as a T-score (for menopausal woman and men older than 50 years) or Z-score (for non-menopausal women and men younger than 50 years) inferior to -1.0 measured by DXA at one or more sites (total femur, femur neck, lumbar spine) or as any radiological evidence of a pathological vertebral fracture. Diuretics use was stopped at least 7 days before the test.

* *P* < 0.05, and

** *P* < 0.001 compared to the overt metabolic acidosis group.

Patients with overt metabolic acidosis (Table 2,column 1) had significantly lower plasma potassium levels, significantly higher fasting urinary pH and lower 24-h urinary citrate excretion than patients without overt metabolic acidosis (Table 2,column 2).

**Table 2:** Biological characteristics of the 67 patients.

	Overt metabolic acidosis	Normal acid-base status
N	11 (16.4%)	56 (83.6%)
Plasma bicarbonate, mmol/L	19.0 [18.0–19.6]	27.0 [25.7–29.0] **
eGFR (MDRD), ml/min/1.73m^2^	65 [56–91]	87 [68–118]
Plasma sodium, mmol/L	136 [135–138]	138 [137–140]
Plasma potassium, mmol/L	3.6 [3.2–3.9]	3.9 [3.7–4.1] *
Serum ionized Ca, mmol/L	1.23 [1.19–1.28]	1.22 [1.19–1.26]
Plasma phosphates, mmol/L	0.95 [0.87–1.02]	0.89 [0.80–1.04]
TmPi/GFR, mmol/L	0.77 [0.38–0.91]	0.85 [0.71–0.96]
Urine fasting pH	6.78 [6.50–6.80]	5.73 [5.47–6.29] **
Urine volume, L/24-h	2.5 [2.0–2.9]	2.03 [1.52–2.39]
Urine creatinine/kg/24-h, mmol/kg/24-h	0.17 [0.13–0.18]	0.18 [0.15–0.21]
Urine citrate, mmol/24-h	**0.060 [0.040–0.113]**	**0.83 [0.58–1.22]** **
Urine NH_4_^+^, mmol/24-h	30.8 [27.7–33.4]	34.8 [27.1–40.1]
Urine fasting Ca/creatinine, mmol/mmol	0.34 [0.11–0.61]	0.27 [0.13–0.37]
Urine Ca, mmol/24-h	3.56 [2.81–5.8]	3.01 [2.26–4.13]
Urine Na, mmol/24h	97 [76–133]	118 [80–144]
Estimated protein intake, g/kg	1.0 [0.8–1.2]	1.0 [0.9–1.2]

Values are expressed as median [interquartile range]. Ca: calcium; eGFR (MDRD): glomerular filtration rate estimated with the Modification of Diet in Renal disease formula; NH4^+^: ammonium; TmPi/GFR: renal phosphate threshold normalized for the glomerular filtration rate. 24-h urines are collected the day before the execution of the acute acid load test; Fasting: patients were kept fasting since midnight of the day before; Urinary fasting results refers to second void morning urines.

* *P* < 0.05, and

** *P* ≤ 0.001 compared to the overt metabolic acidosis group.

Supplemental biological results are reported in Table A of the S1 File.

**Fig 1:**
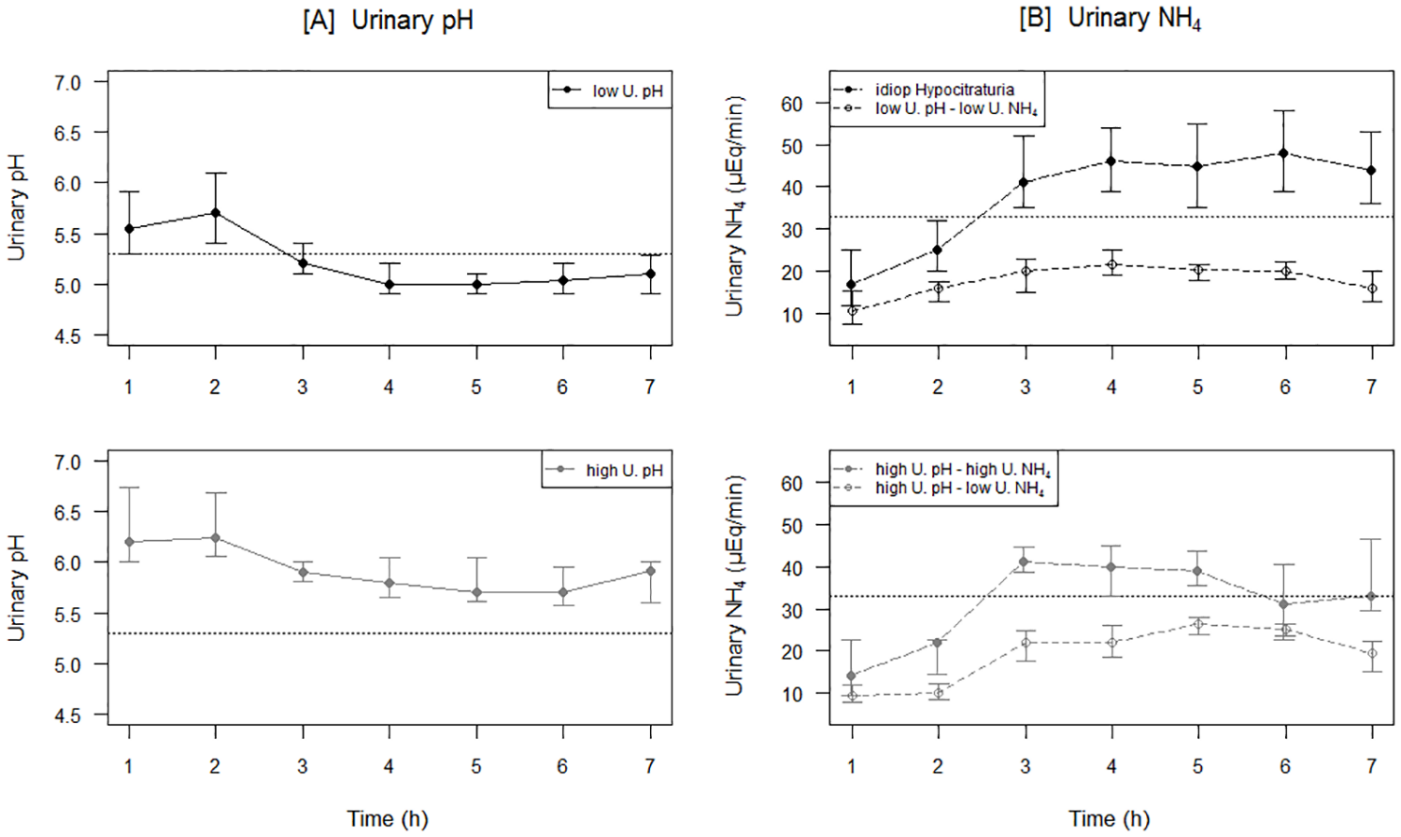
Urinary response to an acute acid load in patients with a normal acid-base status. A) Subdivision in 2 subgroups, according to the ability of the patients to decrease urinary pH below 5.3 (upper panel) or not (lower panel). B) Subdivision into four subgroups according the ability of the subjects to decrease urinary pH below 5.3 and to reach maximal urinary ammonium excretion (U.NH_4_^+^) to 33 μEq/min. Green: idiopathic hypocitraturia defined to both appropriate adaptation of both pH and U.NH_4_^+^). Blue: appropriate maximal urinary ammonium excretion in spite of insufficient urinary acidification (high U. pH, high U.NH_4_^+^ group); Purpura: Inappropriate urinary acidification but appropriate U.NH_4_^+^ (high U. pH, low U.NH_4_^+^ group); Red: Appropriate urinary acidification and U.NH_4_^+^ (low U. pH, low U.NH_4_^+^ group). Points represent the median value, whiskers represent the interquartile range.

The 56 patients with normal plasma HCO_3_^-^ underwent a short NH_4_Cl loading test [4]. The evolution of the urinary pH over time enabled us to split the patients in two groups (Fig 1A): the first group (N = 41) displayed a normal urinary acidification response with pH decreasing <5.3; in the second group (N = 15) urinary pH remained >5.3. Patients were further subdivided according to their ability to excrete acid load, as reflected by the maximal urinary NH_4_^+^-excretion rate (Fig 1B). In 33/56 patients (58.9%), urinary pH decreased <5.3 and urinary NH_4_^+^ excretion rate increased ≥33 μmol/min after an acute acid load. These patients were diagnosed as having idiopathic hypocitraturia. They excreted 15.9% [13.9–17.9] of the acid load within 6 hours, showing an appropriate response to acidification. The other 23 patients (41.1%) had normal baseline plasma HCO_3_^-^ but did not decrease urinary pH <5.3 or did not increase urinary NH_4_^+^ excretion rate ≥33 μmol/min., or both. According to these two criteria, we observed three subtypes of atypical responses to the acid load test. Eleven patients failed to decrease urinary pH but increased urinary NH_4_^+^ to normal levels (the “high U.pH, high U.NH_4_” group). Four patients failed to decrease urinary pH and to increase urinary NH_4_^+^ (the “high U.pH, low U.NH_4_” group). Finally, 8 patients had appropriate decreases in urinary pH but insufficient increase in urinary NH_4_^+^ excretion rate (the “low U.pH, low U.NH_4_” group).

Table 3 shows the baseline characteristics and Table 4 the responses to acute acid load of patients with normal baseline HCO_3_^-^. Significant differences between groups were found in age (higher in "low U.pH, low U.NH_4_"), fasting urinary calcium excretion and 24-hour calcium excretion (higher in "high U.pH, high U.NH_4_"), plasma potassium values (higher in the 2 "low U.NH_4_" subgroups), in eGFR and 24-hour citrate excretion (lower in the 2 "low U.NH_4_" subgroups) and urinary morning pH (lower in "low U.pH, low U.NH_4_"). Importantly, urinary calcium excretion was higher in the "high U.pH, high U.NH_4_” group despite similar natriuresis between groups (Table B of the S1 File).

**Table 3:** Demographic and biological characteristics of the 56 patients with normal baseline plasma HCO_3_^-^ undergoing the acute acid load test.

	Idiopathic Hypocitraturia	High Urine pH High Urine NH_4_^+^	High Urine pH Low Urine NH_4_^+^	Low Urine pH Low Urine NH_4_^+^	*P*
N	33 (58.9%)	11 (19.6%)	4 (7.2%)	8 (14.3%)	
**Age (years)**	**36 [27–44]**	**40 [23–53]**	**46 [40–61]**	**51 [46–68]**	**0.013**
BMI (kg/m^2^)	24.0 [22.1–25.7]	22.4 [18.6–24.8]	21.5 [20.0–23.2]	24.4 [20.2–27.8]	0.236
Gender (female)	14 (42%)	6 (55%)	3 (75%)	3 (38%)	0.557
Plasma bicarbonates, mmol/L	27.0 [25.6–29.0]	27.0 [26.0–29.0]	27.6 [24.7–29.5]	26.6 [25.2–27.7]	0.840
**eGFR (MDRD), mL/min/1.73m**^**2**^	**103 [86–124]**	**78 [69–111]**	**54 [48–72]**	**63 [54–73]**	**<0.001**
Plasma sodium, mmol/L	140 [139–141]	140 [139–142]	140 [138–141]	139 [138–140]	0.843
**Plasma potassium, mmol/L**	**3.9 [3.7–4.1]**	**3.9 [3.6–4.1]**	**4.1 [4.0–4.3]**	**4.2 [3.9–4.4]**	**0.027**
Plasma Chlore, mol/L	103 [101–107]	103.5 [98–105]	103.6 [100–105]	104 [103–105]	0.369
Serum ionized Ca, mmol/L	1.22 [1.19–1.24]	1.22 [1.20–1.27]	1.26 [1.25–1.28]	1.21 [1.19–1.25]	0.186
Urine fasting Ca/creatinine, mmol/mmol*	**0.24 [0.10–0.31]**	**0.41 [0.24–0.47]**	**0.25 [0.21–0.44]**	**0.13 [0.05–0.37]**	**0.046**
**Urine fasting pH**	**5.62 [5.30–6.07]**	**6.20 [5.90–7.10]**	**6.30 [6.10–6.55]**	**5.34 [5.02–5.78]**	**0.005**
**Urine citrate, mmol/24-h**	**1.03 [0.72–1.26]**	**0.67 [0.45–0.99]**	**0.63 [0.41–0.74]**	**0.60 [0.45–0.80]**	**0.021**
Urine NH_4_^+^, mEq/24-h	36.0 [27.9–42.9]	37.6 [29.0–48.2]	27.2 [21.8–32.1]	28.4 [21.0–35.8]	0.141
**Urine Ca, mmol/24-h**	**2.78 [2.00–4.00]**	**4.70 [3.60–4.60]**	**3.10 [2.85–4.21]**	**2.15 [0.89–2.61]**	**0.001**

Values are expressed as median [interquartile range]. BMI: body mass index, Ca: calcium; eGFR (MDRD): glomerular filtration rate estimated with the Modification of Diet in Renal disease formula; NH_4_^+^: ammonium; Fasting: patients were kept fasting since midnight of the day before; Urinary fasting results refers to second void morning urines.

* Upper limit of urine fasting Ca/creatinine = 0.37 mmol/mmol.

Subgroup classifications, according to the minimal urine pH (urine pH min.) and the maximal urine NH_4_^+^ (urine NH_4_^+^ max.) after NH_4_Cl load: **idiopathic hypocitraturia**: min. urine pH < 5.3, max. NH_4_^+^ ≥ 33 μEq/min.; **high U. pH, high U. NH**_**4**_: min. urine pH ≥ 5.3, max. NH_4_^+^ ≥ 33 μEq/min.; **high U. pH, low U. NH**_**4**_: min. urine pH ≥5.3, max. NH_4_^+^ < 33 μEq/min.; **low U. pH, low U. NH**_**4**_: min. urine pH < 5.3, max. NH_4_^+^ < 33 μEq/min. 24-h urines are collected the day before the execution of the acute acid load test.

**Table 4:** Biological results after the acute acid load test of the 56 patients with normal baseline plasma HCO_3_^-^.

	Idiopathic Hypocitraturia	High Urine pH High Urine NH_4_^+^	High Urine pH Low Urine NH_4_^+^	Low Urine pH Low Urine NH_4_^+^	*P*
N	33 (58.9%)	11 (19.6%)	4 (7.2%)	8 (14.3%)	
Plasma bicarbonates min after NH_4_Cl load, mmol/L	22.0 [20.0–25.0]	21.5 [21.0–22.0]	23.0 [22.0–24.0]	23.0 [21.0–28.0]	0.493
**Urine pH min after NH**_**4**_**Cl load**	**5.00 [4.90–5.08]**	**5.63 [5.30–5.90]**	**5.55 [5.45–5.65]**	**4.80 [4.68–5.10]**	**<0.001**
**Urine NH**_**4**_^**+**^ **max. after NH**_**4**_**Cl load, μEq/min**	**53.0 [46.0–62.0]**	**48.0 [41.0–62.0]**	**27.0 [26.5–29.5]**	**25.0 [23.5–26.0]**	**<0.001**
**Urine NAE max. after NH**_**4**_**Cl load, μEq/min**	**76.0 [63.4–90.3]**	**64.5 [50.7–104.4]**	**41.5 [38.4–51.0]**	**43.5 [36.5–48.4]**	**<0.001**
Urine TA max. after NH_4_Cl load, μEq/min	23.0 [18.8–31.0]	25.0 [14.0–43.0]	13.0 [11.0–24.0]	19.5 [12.0–21.0]	0.092

Values are expressed as median [interquartile range]. NAE: net acid excretion; NH_4_^+^: ammonium; NH_4_Cl:ammonium chloride; TA: titrable acidity.

Subgroup classifications, according to the minimal urine pH (urine pH min.) and the maximal urine NH_4_^+^ (urine NH_4_^+^ max.) after NH_4_Cl load: **idiopathic hypocitraturia**: min. urine pH < 5.3, max. NH_4_^+^ ≥ 33 μEq/min.; **high U. pH, high U. NH**_**4**_: min. urine pH ≥ 5.3, max. NH_4_^+^ ≥ 33 μEq/min.; **high U. pH, low U. NH**_**4**_: min. urine pH ≥5.3, max. NH_4_^+^ < 33 μEq/min.; **low U. pH, low U. NH**_**4**_: min. urine pH < 5.3, max. NH_4_^+^ < 33 μEq/min.

Supplemental biological results are reported in Table B of the S1 File.

Of note, urinary citrate excretion overlapped between patients with and without a normal response to the acid load, not allowing defining a threshold to predict the response.

Only one of the 33 patients classified into the idiopathic hypocitraturia group had an eGFR <60 mL/min/1.73 m^2^, compared to 7/23 patients with abnormal response to the acid load (*P* = 0.004), allocated in the “high U.pH, high U.NH_4_” (N = 1), in the “high U.pH, low U.NH_4_” (N = 3) and in the “low U.pH, low U.NH_4_” (N = 3) subgroups.

In multivariate logistic regression including age and protein intake, a normal response (“idiopathic hypocitraturia”) to the acid load was independently associated with higher eGFR (*P* = 0.01) and higher urinary citrate excretion (*P* = 0.04) but no with other characteristics. All groups compounded, the capacity to eliminate the exogenous acid decreased linearly with declining eGFR, both when considering the maximal attained NH_4_^+^-excretion rate (*r* = 0.484,*P*<0.001) and the ratio of the net acid excretion (NAE) divided by NH_4_Cl load (*r* = 0.507,*P*<0.001).

**Fig 2:**
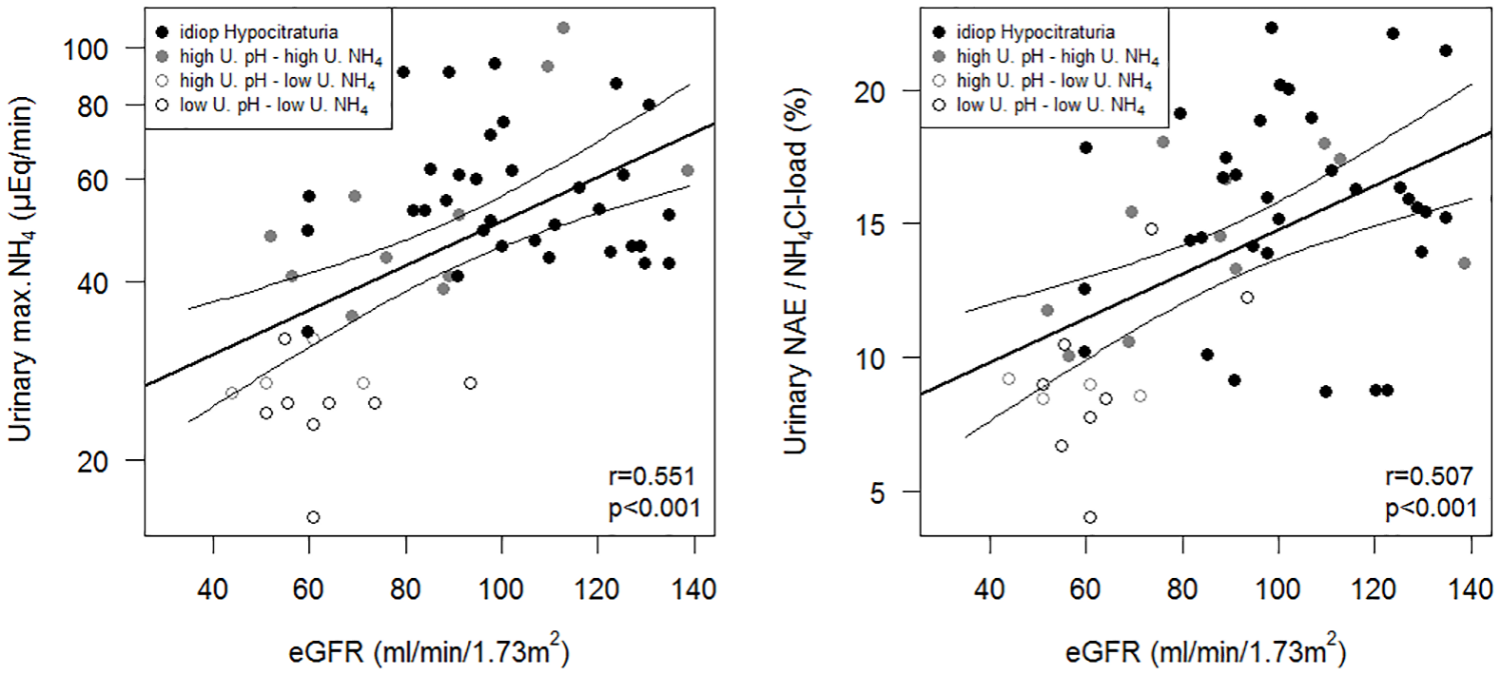
Urinary ammonium excretion rates after acute acid load in patients with a normal acid-base status, according to the urinary acidification defect. All urinary pH values measured within the 6 hours after the acid load are plotted against all the corresponding NH_4_^+^ excretion rates (logarithmic value). Patients were classified in four subgroups according to the minimal pH value and to the maximal NH_4_^+^ excretion rate obtained within this 6 hours: **idiopathic hypocitraturia (green):** min. urine pH < 5.3, max. NH_4_^+^ ≥ 33 μEq/min.; **high U. pH, high U. NH**_**4**_ **(blue):** min. urine pH ≥ 5.3, max. NH_4_^+^ ≥ 33 μEq/min.; **high U. pH, low U. NH**_**4**_ (**purpura):** min. urine pH ≥5.3, max. NH_4_^+^ < 33 μEq/min.; **low U. pH, low U. NH**_**4**_ **(red):** min. urine pH < 5.3, max. NH_4_^+^ < 33 μEq/min. The thick line represents the regression line and the thin lines the 95% confidence intervals of the idiopathic hypocitraturia group (reference group). The horizontal dotted line is the NH_4_^+^ excretion rate cut-off set at 33 μEq/min. The vertical dotted line is the urinary pH cut-off set at 5.3.

To assess interstitial NH_4_^+^ availability, we plotted all NH_4_^+^-excretion results measured during the test as a function of urinary pH (Fig 2). The downward shift of the relationship suggests a decreased interstitial availability in NH_3_ in these patients. When compared to the idiopathic hypocitraturia subgroup, patients with reduced buffer availability had lower NH_4_^+^ values for similar urinary pH values.

### Acidification capacity and related causative diseases

Four out of 11 patients with overt metabolic acidosis (36.4%) had dRTA caused by a Gougerot-Sjögren syndrome. Genetic testing was performed in the seven remaining patients: three patients (42.9%) were heterozygous for the Arg589Cys mutation in the gene encoding AE1 [25]. None of the remaining four patients had mutations in *ATP6V0A4* or *ATP6V1B1* genes or the missense polymorphism (p.Glu161Lys) of the *ATP6V1B1* gene [26] and no cause of dRTA was found in these patients.

**Fig 3:**
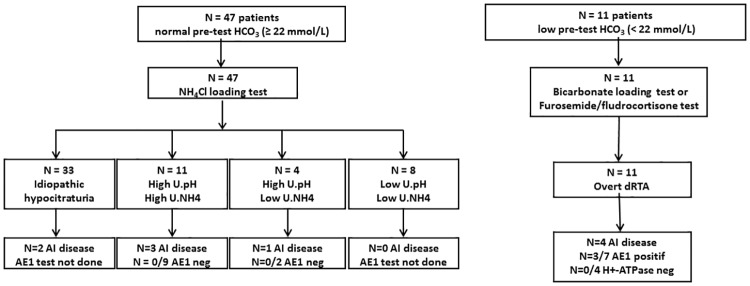
Study results flow chart. Subgroups classifications: **idiopathic hypocitraturia**: min. urine pH < 5.3, max. NH_4_^+^ ≥ 33 μEq/min.; **high U. pH, high U. NH**_**4**_: min. urine pH ≥ 5.3, max. NH_4_^+^ ≥ 33 μmol/min.; **high U. pH, low U. NH**_**4**_: min. urine pH ≥5.3, max. NH_4_^+^ < 33 μEq/min.; **low U. pH, low U. NH**_**4**_: min. urine pH < 5.3, max. NH_4_^+^ < 33 μEq/min. AE1: chloride bicarbonate exchanger; AI: autoimmune disease; E161K: missense polymorphism (p.Glu161Lys) of the ATP6V1B1 gene; H+-ATPase: B1 and a4-subunits of the apical H+-ATPase, including missense polymorphism (p.Glu161Lys) of the *ATP6V1B1* gene; HCO3-: plasma bicarbonate; NH4Cl: ammonium chloride. AI diseases are allocated as follows: **"idiopathic hypocitraturia"**: spondyloarthritis (N = 1), rheumatic polyarthritis (N = 1); "**high U.pH, high U.NH4"**: Gougerot-Sjögren disease (N = 1), spondylarthritis (N = 1), primary biliary cirrhosis (N = 1); **"high U.pH, low U.NH**_**4**_^**+**^**"**: Crohn’s disease (N = 1).

Six subjects (10.7%) with normal baseline HCO_3_^-^ had an autoimmune disease (Fig 3). Genetic testing was accepted by 10/11 patients (90.9%) with a normal acid-base status who were unable to decrease the urinary pH<5.3 and without autoimmune disease. These patients had neither mutations in *SLC4A1* (Fig 3) nor the previously described *ATP6V1B1* polymorphism [26].

## Discussion

In our investigations of patients with hypocitraturia, underlying conditions were overt dRTA (16.4%), idiopathic hypocitraturia (49.3%), and subtle abnormalities in response to acid load (34.3%). The latter group was subdivided on the bases of urinary pH and NH_4_^+^-excretion in response to an acute acid load.

In patients with overt dRTA, etiologic screening was profitable, with a genetic cause being identified in 42.9% of cases (N = 3) without autoimmune disease (N = 7), all having the same missense mutation in Arg589 of the *SLC4A1* gene, common in Europe [6, 25]. Four were diagnosed with Gougerot-Sjögren syndrome. In the remaining 4 cases, no genetic/autoimmune cause was found. We cannot exclude that they will manifest in the future an autoimmune disease or that they have a predisposing genetic mutation, not yet identified.

Patients with normal baseline acid-base status were challenged with acute acid load test. Urine pH may be misleading when trying to assess the integrity of the distal urinary acidification, since it measures only the activity of free hydrogens ions, which is less than 1% of the total amount of protons excreted. In the presence of a high amount of buffer in the urine, as observed in patients with chronic extrarenal acidosis, urinary pH may remain >5.3 despite appropriate acid excretion. Conversely, in the presence of low buffer availability, an apparent appropriate low urine pH does not exclude a defect in net acid excretion [27]. We thus took both parameters into account.

Of patients with normal acid-base status under basal conditions, 58.9% had idiopathic hypocitraturia (normal evolution of urinary pH and NH_4_^+^ after acute acid load). Excepted for low urinary citrate excretion, the biological profiles of these patients were within the normal ranges. We found no easily available adjunctive element that allows prediction of the response to an acid challenge test, with the exception of an impaired eGFR (<60 mL/min/1.73 m^2^), which is virtually always predictive of an abnormal response to the acid load test.

Among patients with a normal acid-base status, 41.1% were either unable to decrease urinary pH <5.3 and/or to increase NH_4_^+^-excretion rate ≥33 μEq/min. when challenged with an acid load. The pathophysiological signification of these defects is in question. In 1959, Wrong [4] described the cases of three patients with nephrocalcinosis, who were unable to decrease their urinary pH after an acute acid challenge. Some of these patients displayed an appropriate response in terms of NH_4_^+^ and NAE, suggesting that NH_3_/NH_4_^+^ accumulates in the renal interstitium. In line with this, 11/56 patients (19.6%) in our study with normal basal acid-base status failed to appropriately acidify their urine after the acute acid load, although they did show an appropriate increase in urinary NH_4_^+^. The relevance of this defect that does not impair NAE is questionable. This abnormality could reflect increased ammoniogenesis due either to a primary event in proximal tubule [4, 28] or to subtle extrarenal bicarbonate loss/acid load that may chronically stimulate ammoniogenesis, and explain the low urinary citrate excretion. Whatever the underlying mechanism, it has been proposed that the resulting accumulation in medullary interstitium of NH_3_/NH_4_^+^ might be deleterious for the kidney [28, 29]. These patients had however conserved eGFR and showed no increase in 24-hour urinary NH_4_^+^-excretion, suggesting no extrarenal acidosis. Interestingly, this group had a higher 24-hour urinary calcium excretion than all other subgroups, as well as a higher urinary fasting calcium/creatinine ratio (median of 0.41 mmol/mmol for a upper limit of 0.37 mmol/mmol), reflecting higher net bone resorption (fasting calcium/creatinine ratio is measured after 12-hours fasting; the calcium measured with this test comes mainly from bone and reflects net bone resorption, i.e. the difference between the rates of mineralization and resorption). These findings suggest that this form of urinary acidification dysregulation may have deleterious repercussions on bone and kidney. Of note, higher urinary calcium excretion in this group was not due to higher sodium intake since 24-hour natriuresis was similar in all groups. Even if these patients are not considered to have abnormal adaptation to acid load, since they were able to adapt their acid rate excretion, it is worthy of further investigation to determine whether these patients have extrarenal disease or primary proximal tubular defects. Due to the deleterious effect of NH_3_/NH_4_^+^ on kidney interstitium [28], this early presentation with “high U.pH, high U.NH_4_” could switch to a later presentation with lower GFR and lower availability in interstitial NH_3_/NH_4_^+^, such as observed in the “low U.pH, low U.NH_4_” group and illustrated by the relationship between pH and NH_4_^+^ excretion.

When challenged with the acid load, 8/56 patients (14.3%) were able to adapt their urinary pH but not their NH_4_^+^ excretion rate. These patients are systematically missed when considering only the urinary pH response to the acid load. A low NH_4_^+^ excretion may be due to a blunted ammoniogenesis in the proximal tubule by the metabolism of glutamine or to a NH_3_ transfer defect through the renal interstitium to the collecting duct lumen, the latter being explained for example by an interstitial disease (impaired countercurrent system) or by hyperkalemia [27]. None of our patients presented with hyperkalemia, but patients of this subgroup were older and their eGFRs were lower (more than expected considering physiologic loss of GFR with aging) than in patients with idiopathic hypocitraturia. The capacity of these patients to eliminate the acid load was almost halved when compared to patients with normal renal acid handling.

Finally, 4/56 patients (7.2%) were unable to adapt both their urinary pH and NH_4_^+^-excretion rate to the acid load, defining a renal acidification defect usually referred as incomplete dRTA.

Regarding the etiologic screening of patients with a masked urinary acidification defect, the prevalence of an autoimmune disorder was 17% (vs. 36% in the overt dRTA group) and included several types of autoimmune disease. We suggest screening for symptoms indicative of a systemic immunologic disease, based on previous demonstrations of the relationships of several autoimmune diseases with acquired urinary acidification defects [9, 10]. Some case series demonstrated dominant *SLC4A1* mutations in patients with a masked acidification defect [25, 30] or the presence of the missense polymorphism (p.Glu161Lys) of the *ATP6V1B1* gene [26]. In our series, neither mutations in the *SLC4A1* gene nor this polymorphism were detected in hypocitraturic patients with normal basal acid-base status and altered urinary acidification capacity.

The main question is whether characterization of the masked urinary acidification defect would influence the management of the patients. In patients with alkaline urine and preserved ammoniogenesis, the administration of citrate is the priority, as this treatment corrects intracellular acidosis. Thiazide diuretics, used to decrease hypercalciuria, should be carefully titrated to avoid hypokalemia and aggravation of hypocitraturia [3]. Whether citrate treatment is sufficient (without the use of thiazide diuretics) to normalize fasting calciuria deserves a dedicated study. In patients with reduced availability of NH_4_^+^, the therapy should focus on the reason for lack of buffer (i.e., impaired ammoniogenesis or defects in ammonium transfer through the medullary interstitium) with correction of hyperkalemia if present. The therapeutic approach should induce reductions in the exogenous acid load (e.g., animal protein intake). The prescription of potassium citrate should be considered, but plasma potassium should carefully be monitored if renal function is impaired.

Our study has several limitations. Because of its retrospective design, we could not determine whether the masked phenotype (with normal baseline plasma HCO3^-^) progresses to the overt metabolic acidosis phenotype or whether there is a continuum that bridges the different subgroups with subtle urinary acidification defects. Concerning the etiologic screening, biomarkers for autoimmune diseases were investigated only in presence of clinical signs. Thus, the real prevalence of an autoimmune disorder cannot be formally assessed, and a random association cannot be excluded. Screening for *SLC4A1* mutations was refused by three patients with a masked urinary acidification defect; therefore, we cannot exclude the occurrence of a hereditary cause in patients presenting with normal HCO_3_^-^. DXA was not systematically performed. Consequently, we could not assess the impact of a latent urinary acidification defect on bone mineralization.

We did not repeat acid load in our patients since we conducted a retrospective study including all the patients explored in our department due to hypocitraturia. Of note, the acidification test takes time (usually more than 6 hours) and cannot be repeated easily. Given the complexity and the length of this exploration, this question would require a dedicated prospective study, which has never been performed before to our best knowledge. However, in healthy volunteers previously explored in our department for another work, we could check that the inter-individual variability of the response to the acidification test was very low suggesting a low intra-individual variability for a given patient.

## Conclusions

In conclusion, in hypocitraturic patients with normal plasma HCO_3_^-^, an accurate diagnosis of a masked urinary acidification defect requires a functional acid challenge test, which should consider both urinary NH_4_^+^ and urinary pH response. Indeed, in a significant number of patients, inappropriately high urinary pH was associated with appropriate adaptation of NH_4_^+^ excretion. We have shown that these patients should be screened for high urinary fasting calcium excretion and should benefit from further investigation to determine whether they have extrarenal disease or primary proximal tubular defects. Conversely, we have highlighted that apparently appropriate low urinary pH could be associated with blunted adaptation in NH_4_^+^ excretion. Even if these patients were until now theoretically not considered to have abnormal adaptation to acid load, we have shown that these patients were at risk of having low GFR.,

Consequently, the precise characterization of the biological phenotype underlying hypocitraturia is of high importance to adapt etiologic screening and therapeutic management to optimize the care of patients with nephrolithiasis.

## Supporting information

S1 File Additional method’s details. **Table A. Biological characteristics of the patients, according to the basal abnormalities in the acid-base status.** Values are expressed as median [interquartile range]. Ca^2+^: calcium; P: plasma. * *p* < 0.05 compared to the overt metabolic acidosis group. **Table B. Biological characteristics of patients with a normal acid-base status undergoing the acute acid load test.** Values are expressed as median [interquartile range]. Ca^2+^: calcium; NH_4_^+^: ammonium; P: plasma; NH_4_Cl: ammonium chloride. Subgroups classification: Idiopathic Hypocitraturia: urine pH min < 5.3, NH_4_^+^ max ≥ 33 μEq/min.; high U.pH, high U.NH4: urine pH min < 5.3, NH_4_^+^ max ≥ 33 μEq/min.; high U.pH, low U.NH_4_: urine pH min ≥5.3, NH_4_^+^ max ≥ 33 μEq/min.; low U.pH, low U.NH_4_: urine pH min < 5.3, NH_4_^+^ max < 33 μEq/min.
(DOCX)
