# Supplementary material for: Signification of distal urinary acidification defects in hypocitraturic patients
Source: PLoS One. 2017 May 19;12(5):e0177329. doi: 10.1371/journal.pone.0177329 (PMC5438111; doi:10.1371/journal.pone.0177329)
Supplement: S1 File — Table A. Biological characteristics of the patients, according to the basal abnormalities in the acid-base status. Values are expressed as median [interquartile range]. Ca2+: calcium; P: plasma. * p < 0.05 compared to the overt metabolic acidosis group. Table B. Biological characteristics of patients with a normal acid-base status undergoing the acute acid load test. Values are expressed as median [interquartile range]. Ca2+: calcium; NH4+: ammonium; P: plasma; NH4Cl: ammonium chloride. Subgroups classification: Idiopathic Hypocitraturia: urine pH min < 5.3, NH4+ max ≥ 33 μEq/min.; high U.pH, high U.NH4: urine pH min < 5.3, NH4+ max ≥ 33 μEq/min.; high U.pH, low U.NH4: urine pH min ≥5.3, NH4+ max ≥ 33 μEq/min.; low U.pH, low U.NH4: urine pH min < 5.3, NH4+ max < 33 μEq/min. (DOCX) [file pone.0177329.s001.docx]

**Supporting Information**

**Additional Methods’ details**

**Bicarbonate loading test**

Patients were challenged with IV infusion of 1.4% sodium bicarbonate at a rate of 300 ml/h. Venous blood was drawn through an indwelling catheter for measurement of pH and pCO_2_ at baseline and at hourly intervals thereafter during 4 hours. In alkaline urine, such as after a load of sodium bicarbonate, urine PCO_2_ increases because of distal H^+^ secretion, which reacts with luminal HCO_3_^-^ to form carbonic acid and dehydrates slowly in the medullary collecting duct to form CO_2_ that is trapped in the renal tubule. When urine HCO_3_^-^ concentration is higher than plasma HCO_3_^-^ concentration, the urine-to-blood PCO_2_ gradient should be > 20 mmHg. Patients with decreased rates of distal tubular H^+^ secretion show subnormal values, with urine-to-blood PCO_2_ gradient ≤ 20 mmHg [1].

**Furosemide/fludrocortisone test**

After plasma and urine sampling, we simultaneously administered oral furosemide 40 mg and fludrocortisone 1 mg. Plasma and urine were collected hourly for 6 hours after the baseline sample. A distal acidification defect was identified by a failure to lower the urinary pH < 5.3 and/or to increase NH_4_^+^ excretion rate ≥ 33 µmol/min.

The first period (1 hour) was designated as the control period. Then, the subjects were challenged with oral furosemide 40 mg and oral fludrocortisone 1 mg. At the end of each timed interval of 1 hour over a period of 6 hours, a urine sample for pH, pCO_2_, and concentrations of ammonium (NH_4_), titrable acid (TA) was collected. Venous blood was drawn through an indwelling catheter for measurement of pH and pCO_2_ at baseline and at hourly intervals thereafter. The increase in hourly diuresis, natriuresis, and kaliuresis following furosemide/fludrocortisone administration was verified to ensure the validity of the test.

**Acute acid load**

Oral administration of 2 mmol/kg body weight ammonium chloride (NH_4_Cl) was performed after plasma and urine sampling [2]. Inability to lower urine pH < 5.3 and/or to increase NH_4_^+^ excretion rate ≥ 33 µEq/min. during 6 consecutive hours identified the urinary acidification defect. The first period (1 hour) was designated as the control period. Afterwards, the subjects were given orally the acid load (ammonium chloride 2 mmol/kg). At the end of each timed interval of 1 hour over a period of 6 hours, a urine sample for pH, pCO_2_, and concentrations of ammonium (NH_4_), titrable acid (TA) was collected. Venous blood was drawn through an indwelling catheter for measurement of pH and pCO_2_ at baseline and at hourly intervals thereafter. Only subjects with a valid NH_4_Cl test, defined by a plasma pH decrease ˃0.06 and/or a plasma HCO_3_ concentration variation of -4.1 ± 1.5 mmol/L after acid challenge were included for the present analysis.

**Analytical methods**

Estimated glomerular filtration rate (eGFR) was obtained using the Modification of Renal Disease (MDRD) equation [6]. The 24-h urea nitrogen, measured by urease–GLDH method, was used to estimate daily protein intake, as proposed by Maroni *et al.* [7]. The renal phosphate threshold (TmPi/GFR) was calculated using the nomogram by Bijvoët [8].

Plasma concentrations of sodium, potassium, total calcium, ionized calcium, phosphorus, magnesium, creatinine, uric acid, 25(OH)-vitamin D, 1,25(OH)_2_-vitamin D, intact parathyroid hormone (PTH), were measured at baseline. Serum and urine creatinine were measured using the kinetic colorimetric compensated Jaffe method. Ionized calcium was determined using a calcium ion-selective electrode (Analyzer ABL 555 then 705, Radiometer, Copenhagen, Denmark). Serum and urine concentrations of sodium, potassium, calcium, and magnesium were measured by atomic absorption spectrometry, phosphorus by phosphomolybdate UV colorimetric method, chloride by potentiometer, uric acid and citrate by enzymatic methods, plasma intact PTH by sandwich second generation methods, and plasma 25(OH)-vitamin D and 1,25 (OH)_2_-vitamin D by competitive RIA methods. All data were analyzed in accordance with reference ranges for each method.

**Dual-energy X-ray absorptiometry (DXA)**

According to the criteria proposed by the World Health Organization (WHO), bone demineralization was defined as an abnormal reduced bone mineral density, defined by a T-score (for menopausal women and men older than 50 years) or Z-score (for non-menopausal women and men younger than 50 years) inferior to -1.0 measured by DXA at one or more sites (total femur, femur neck, lumbar spine) [9]. Any radiological evidence of a pathological vertebral fracture was also taken as a diagnostic criterion of bone demineralization.

**DNA sequence analysis**

DNA was extracted with a high-salt method or with blood DNA midi kits (Qiagen columns). *SLC4A1* exons and the flanking intron sequences were amplified by PCR and then sequenced with BigDye Terminator kit v3.1 cycle sequencing kits and run on an ABI Prism 3730XL DNA Analyzer Sequencer (Perkin Elmer Applied Biosystems, Foster City, CA). The primers and PCR conditions used for *ATP6V0A4* and *ATP6V1B1* have been previously described [10], including the search of the missense p.Glu161Lys polymorphism of the exon 6 of the ATP6V1B1 gene [11]. For *SLC4A1,* the primers and PCR conditions used are described in following Table.

| Exon | Forward (5'-3') | Reverse (5'-3') | Tm | Product length (bp) |
| --- | --- | --- | --- | --- |
| 2 and 3 | TCG CTG GTG CCA GCC AGA CTT | TGC CCG CCC TTC TCA CAT TGC | 66°C | 776 |
| 4 | TGT GGG ATG CGG CAC CAG | ATC TCT GCC CAG GGT GCT CCT | 66°C | 421 |
| 5 | GGA TCC AAG CCT CAC AAG | ACA GCA CCC CAC AAC AAT | 52°C | 330 |
| 6 | TGC AGA TGA GGA TTG TTG | CCC CAA CTC TGA GCA TAG | 52°C | 391 |
| 7 | CCA GTC CCT TGA TGT TCG | CTT CCC ATT CTC GCT TCC | 52°C | 376 |
| 8 | GAG AGT TGG GAA GCG AGA A | TGT GGA GGG CTG AGG GTA GA | 62°C | 214 |
| 9 | CCC CCG CAG CCC CAT CTC TT | GCC CCC GCC AGG TAG GAT AG | 62°C | 408 |
| 10 | TGG AGG CCC TGG CTG TGG ACT | TCG GTG GGG GCT CAG AAA G | 62°C | 331 |
| 11 | CCC CAT TCC CAT CAG ACA A | TTC CAG GAG CCC ATA GAG C | 62°C | 430 |
| 12 and 13 | CTA TGG GCT CCT GGA AAT GA | TCG GCC TCC CAA AGT TCT GA | 62°C | 624 |
| 14 | GAA TGA TGG ATG GGT GGA TA | GCT TGC CCA TAG AGT GAA AC | 62°C | 494 |
| 15 | TAT AGG GCA GGA GGA AGG CA | TGG GGG GTG GAA ATG AGG AC | 62°C | 223 |
| 16 | TCC CAA GTG CCT CCA ACC TA | GCC CCT GGC TTT TCA CTA TT | 62°C | 432 |
| 17 | GAG GGG CAC AGA AGA CAA AA | TGG GGA AGT GGT GCA GGA T | 62°C | 491 |
| 18 | ATT CCC AGC CCC AGA TAG | GGA TGC CCG TGA ATA AGT G | 62°C | 378 |
| 19 | TAC GTC AAG CGG GTA CAG GAC | AGC CCT AGC CCC AGA CTT TA | 62°C | 381 |
| 20 | TTC AAG ACA GCC AGA CCC TC | GAG GTG CCC ATG AAC TTC TG | 62°C | 274 |

DNA mutations in the *SLC4A1*gene were identified with Sequencher software, through comparisons with the reference sequence: NM_000342.3. Each mutation was confirmed by sequencing a second independent PCR product.

**References**

1. DuBose TD, Jr., Caflisch CR. Validation of the difference in urine and blood carbon dioxide tension

during bicarbonate loading as an index of distal nephron acidification in experimental models of distal renal tubular acidosis. The Journal of clinical investigation. 1985;75(4):1116-23.

2. Wrong O, Davies HE. The excretion of acid in renal disease. Q J Med. 1959;28(110):259-313.

3. Jorgensen K. Titrimetric determination of the net excretion of acid/base in urine. Scand J Clin Lab Invest 1987(9):287-91.

4. Nutbourne DM. The effect of dilution on the titratable acid in urine and acidified phosphate buffer solutions, and the correction for this effect in the determination of the rate of elimination of hydrogen ions from the body by the renal tubules. Clin Sci. 1961;20:263-78.

5. Elkinton JR, Huth EJ, Webster GD, Jr., Mc CR. The renal excretion of hydrogen ion in renal tubular acidosis. I. quantitative assessment of the response to ammonium chloride as an acid load. Am J Med. 1960;29:554-75.

6. Levey AS, Coresh J, Greene T, Stevens LA, Zhang YL, Hendriksen S, et al. Using standardized serum creatinine values in the modification of diet in renal disease study equation for estimating glomerular filtration rate. Annals of internal medicine. 2006;145(4):247-54.

7. Maroni BJ, Steinman TI, Mitch WE. A method for estimating nitrogen intake of patients with chronic renal failure. Kidney Int. 1985;27(1):58-65.

8. Walton RJ, Bijvoet OL. Nomogram for derivation of renal threshold phosphate concentration. Lancet. 1975;2(7929):309-10.

9. Assessment of fracture risk and its application to screening for postmenopausal osteoporosis. Report of a WHO Study Group. World Health Organ Tech Rep Ser. 1994;843:1-129.

10. Vargas-Poussou R, Houillier P, Le Pottier N, Strompf L, Loirat C, Baudouin V, et al. Genetic investigation of autosomal recessive distal renal tubular acidosis: evidence for early sensorineural hearing loss associated with mutations in the ATP6V0A4 gene. J Am Soc Nephrol. 2006;17(5):1437-43.

11. Dhayat NA, Schaller A, Albano G, Poindexter J, Griffith C, Pasch A, et al. The Vacuolar H+-ATPase B1 Subunit Polymorphism p.E161K Associates with Impaired Urinary Acidification in Recurrent Stone Formers. J Am Soc Nephrol. 2015.

**Table A. Supplemental biological characteristics of the patients, according to the basal abnormalities in the acid-base status.**

|  | **Overt metabolic acidosis** | **Normal acid-base status** |
| --- | --- | --- |
| N | 11 | 56 |
| P. Chlore, mmol/L | 106 [104 - 110] | 103 [102 - 104] |
| P. Uric acid, µmol/L | 254 [225 - 288] | 321 [246 - 364] |
| P. total Ca^2+^, mmol/L | 2.29 [2.18 - 2.32] | 2.24 [2.19 - 2.29] |
| P. iPTH, pg/mL | 29 [18 - 53] | 40 [28 - 52] |
| P. 25(OH) Vit. D, nmol/L | 38 [30 - 76] | 57 [42 - 86] |
| **P. 1,25(OH) Vit. D, pmol/L** | **93 [56 - 121]** | **133 [110 - 163] *** |

Values Values are expressed as median [interquartile range]. Ca^2+^: calcium; P: plasma.

* *p* < 0.05 compared to the overt metabolic acidosis group.

**Table B. Supplemental biological characteristics of patients with a normal acid-base status undergoing the acute acid load test.**

|  | **Idiopatic**  **Hypocitraturia** | **High U. pH**  **High U. NH_4_** | **High U. pH**  **Low U. NH_4_** | **Low U. pH**  **Low U. NH_4_** | **P** |
| --- | --- | --- | --- | --- | --- |
| N | 33 | 11 | 4 | 8 |  |
| Plasma total Ca^2+^, mmol/L | 2.21 [2.18 - 2.27] | 2.24 [2.21-2.40] | 2.25 [2.20-2.30] | 2.25 [2.19-2.29] | 0.464 |
| Plasma phosphates,  mmol/L | 0.88 [0.79 - 1.04] | 1.02 [0.88-1.06] | 0.84 [0.78-0.93] | 0.93 [0.87-1.00] | 0.416 |
| TmPi/GFR, mmol/L | 0.89 [0.74 - 0.97] | 0.91 [0.74-0.99] | 0.71 [0.64-0.74] | 0.76 [0.68-0.93] | 0.129 |
| Plasma iPTH, pg/mL | 38 [31 - 52] | 27 [21 - 49] | 34 [20 - 45] | 43 [40 - 64] | 0.323 |
| Plasma 25(OH) Vit. D, mol/L | 51 [41 - 80] | 70 [42 - 95] | 56 [48 - 80] | 72 [46 - 92] | 0.551 |
| Plasma 1,25(OH) Vit. D, mol/L | 137 [111 - 167] | 142 [102 - 209] | 163 [97 - 165] | 114 [74 - 132] | 0.335 |
| Urine volume, L/24-h | 2.00 [1.40 - 2.34] | 2.24 [1.70-3.40] | 2.14 [1.91-2.29] | 1.77 1.53-2.24] | 0.208 |
| Urine creatinine,  mmol/kg/24-h | 0.19 [0.15 - 0.21] | 0.18 [0.17-0.23] | 0.17 [0.15-0.19] | 0.17 [0.13-0.17] | 0.337 |
| Urine Na, mmol/24-h | 149 [95 - 181] | 137 [109 - 212] | 85 [78 - 89] | 136 [92 - 177] | 0.109 |

Values are expressed as median [interquartile range]. Ca^2+^: calcium; NH_4_^+^ : ammonium; NH_4_Cl : ammonium chloride; Na = sodium; TmPi/GFR: renal phosphate threshold normalized for the glomerular filtration rate. Subgroups classification: Idiopathic Hypocitraturia: urine pH min < 5.3, NH_4_^+^ max ≥ 33 µEq/min.; high U.pH, high U.NH4: urine pH min < 5.3, NH_4_^+^ max ≥ 33 µEq/min.; high U.pH, low U.NH_4_ : urine pH min ≥5.3, NH_4_^+^ max ≥ 33 µEq/min.; low U.pH, low U.NH_4_ : urine pH min < 5.3, NH_4_^+^ max < 33 µE
